# Binding of *Plasmodium falciparum* Merozoite Surface Proteins DBLMSP and DBLMSP2 to Human Immunoglobulin M Is Conserved among Broadly Diverged Sequence Variants*[Fn FN2]

**DOI:** 10.1074/jbc.M116.722074

**Published:** 2016-05-12

**Authors:** Cécile Crosnier, Zamin Iqbal, Ellen Knuepfer, Sorina Maciuca, Abigail J. Perrin, Gathoni Kamuyu, David Goulding, Leyla Y. Bustamante, Alistair Miles, Shona C. Moore, Gordon Dougan, Anthony A. Holder, Dominic P. Kwiatkowski, Julian C. Rayner, Richard J. Pleass, Gavin J. Wright

**Affiliations:** From the ‡ Cell Surface Signalling Laboratory,; the § Malaria Programme, and; the ** Microbial Pathogenesis Laboratory, Wellcome Trust Sanger Institute, Cambridge CB10 1SA, United Kingdom,; the ¶ Wellcome Trust Centre for Human Genetics, Oxford OX3 7BN, United Kingdom,; the ‖ Francis Crick Institute, Mill Hill Laboratory, London NW7 1AA, United Kingdom,; the ‡‡ Department of Parasitology, Liverpool School of Tropical Medicine, Liverpool L3 5QA, United Kingdom, and; the §§ Warwick Systems Biology Centre, Senate House, University of Warwick, Coventry CV4 7AL, United Kingdom

**Keywords:** genetic polymorphism, host-pathogen interaction, parasite, plasmodium, protein-protein interaction, immunoglobulin M (IgM), merozoite

## Abstract

Diversity at pathogen genetic loci can be driven by host adaptive immune selection pressure and may reveal proteins important for parasite biology. Population-based genome sequencing of *Plasmodium falciparum*, the parasite responsible for the most severe form of malaria, has highlighted two related polymorphic genes called *dblmsp* and *dblmsp2*, which encode Duffy binding-like (DBL) domain-containing proteins located on the merozoite surface but whose function remains unknown. Using recombinant proteins and transgenic parasites, we show that DBLMSP and DBLMSP2 directly and avidly bind human IgM via their DBL domains. We used whole genome sequence data from over 400 African and Asian *P. falciparum* isolates to show that *dblmsp* and *dblmsp2* exhibit extreme protein polymorphism in their DBL domain, with multiple variants of two major allelic classes present in every population tested. Despite this variability, the IgM binding function was retained across diverse sequence representatives. Although this interaction did not seem to have an effect on the ability of the parasite to invade red blood cells, binding of DBLMSP and DBLMSP2 to IgM inhibited the overall immunoreactivity of these proteins to IgG from patients who had been exposed to the parasite. This suggests that IgM binding might mask these proteins from the host humoral immune system.

## Introduction

Malaria is responsible for up to one million deaths annually and therefore remains one of the world's major health problems (1, 2). Among the different species of *Plasmodium* causing malaria in humans, *Plasmodium falciparum* is responsible for the highest burden of disease. The clinical symptoms of malaria are associated with the blood stage of the infection, when the merozoite stage of the parasite recognizes, invades, and develops within human erythrocytes (3). Because merozoites are directly exposed to circulating antibodies and passive immunization of infected children with purified immunoglobulins from clinically immune individuals reduces parasitemia (4), merozoite surface proteins are considered likely targets of host immunity and therefore potential vaccine candidates (5). Consistent with this, population-based *P. falciparum* genome sequence analysis (6) has revealed that genes encoding merozoite surface proteins are among the most polymorphic in the genome, with several exhibiting signatures of balancing selection, suggesting that host immune pressure maintains the presence of multiple distinct antigenic variants (7).

*dblmsp* and *dblmsp2* (also known as *msp3.4* and *msp3.8*, respectively) are two members of the *P. falciparum msp3* family that comprises a cluster of eight paralogous genes on chromosome 10. The *msp3* family encodes proteins, which are characterized by the presence of an NLR(K/N)(A/G/N) motif at their N terminus (8, 9), are secreted by the blood stage of the parasite, and located in both the parasitophorous vacuole and on the merozoite surface. Most proteins within this family also contain a C-terminal acidic and a coiled-coil region believed to be involved in oligomerization of the proteins (10), which form the SPAM (secreted polymorphic antigen associated with merozoites) domain (11). DBLMSP and DBLMSP2 are distinguished from the other MSP3 family members because they contain a Duffy binding-like (DBL)^5^ domain (8, 12). DBL domains are known to bind directly to host receptors and are present in other *P. falciparum* surface proteins including ligands involved in erythrocyte invasion such as EBA175 and EBA140 (13–15) and members of the PfEMP1 family, which are displayed on the surface of the infected erythrocyte (16). Sequencing of some African *P. falciparum* strains has shown that the genetic diversity in *dblmsp* and *dblmsp2* is particularly high and concentrated in the region encoding the DBL domain (17–19). Clearly identifiable orthologs of *dblmsp* and *dblmsp2* are not present in other *Plasmodium* species that infect humans, but the genome of the chimpanzee parasite *Plasmodium reichenowi* encodes a functional *dblmsp* gene, although *dblmsp2* is a pseudogene (19). Despite these interesting features, the function of DBLMSP and DBLMSP2 and their role in the pathology of malaria are unknown.

Here, we used recombinant proteins produced in mammalian cells and a knock-out parasite line to show that the DBL domains of DBLMSP and DBLMSP2 from the 3D7 strain of *P. falciparum* bind avidly and directly to human IgM. Using population-based genome sequencing and bespoke assembly tools, we revealed widespread genetic diversity focused on the DBL domain, with two major allelic forms of both *dblmsp* and *dblmsp2* observed in populations from Africa and Southeast Asia. Despite their diversity, binding of the DBL domains from different DBLMSP and DBLMSP2 sequence variants to human IgM was conserved, suggesting an important role in the parasite biology.

## Results

### 

#### 

##### DBLMSP and DBLMSP2 Bind Human IgM

**FIGURE 1.**
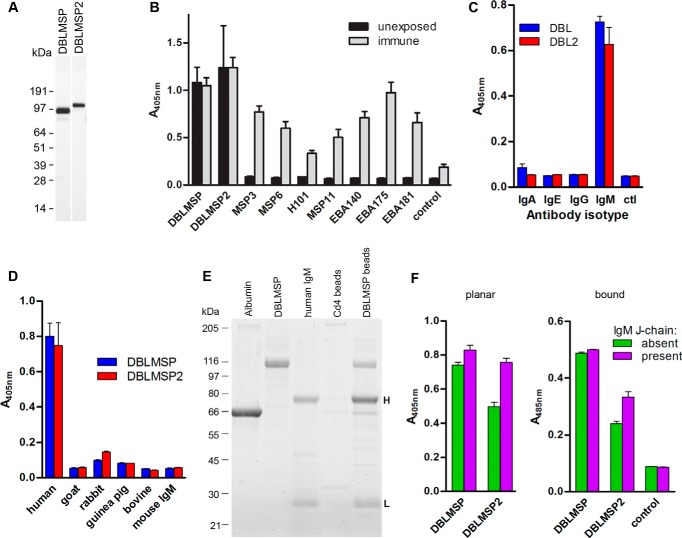
**Recombinant *P. falciparum* DBLMSP and DBLMSP2 bind human IgM.** *A*, anti-biotin Western blot showing expression of recombinant monobiotinylated DBLMSP and DBLMSP2 proteins from mammalian cells. *B*, DBLMSP and DBLMSP2 are immunoreactive with control, naïve human sera. Enzymatically monobiotinylated recombinant parasite proteins were immobilized on streptavidin-coated plates, and their immunoreactivity to pooled sera from either unexposed individuals (*black bars*) or immune Kenyan adults (*gray bars*) was tested. *C*, DBLMSP (*blue*) and DBLMSP2 (*red*) bind purified human IgM and no other immunoglobulin isotype, relative to no immunoglobulin control (*ctl*). *D*, DBLMSP and DBLMSP2 binding was restricted to human IgM, and did not bind sera from other species, including purified murine IgM. *E*, streptavidin-coated paramagnetic beads were coated in monobiotinylated His_6_-tagged recombinant DBLMSP or a control protein (Cd4-d3 + 4) and incubated in the presence of normal human serum. Following elution and resolution by SDS-PAGE, 80- and 25-kDa bands corresponding to the heavy (*H*) and light (*L*) chains of human IgM, respectively, were observed in the DBLMSP but not the negative control pulldown. Purified albumin, DBLMSP, and IgM protein are shown for comparison on the *left. F*, both DBLMSP and DBLMSP2 bound chimeric IgM containing human heavy chains regardless of whether it was antigen-bound (*right panel*) or not (*left panel*). Both DBLMSP and DBLMSP2 could bind IgM that contained or lacked a J-chain, although DBLMSP2 binding to IgM lacking a J-chain was reproducibly weaker. Negative control was the Cd4 tag alone. *B–D* and *F* represent one representative from two or more ELISA experiments; *bars* represent means ± S.D.; *n* = 3 replicate wells.

To gain insight into the functional role of DBLMSP and DBLMSP2 during malaria pathogenesis, we first expressed the entire coding region of both proteins from the *P. falciparum* 3D7 strain using a recently developed expression system based on mammalian cells, which has been shown to produce natively folded *P. falciparum* proteins (20). Both proteins were expressed (Fig. 1*A*), and their immunoreactivity to sera from malaria-exposed and unexposed control individuals was quantified. To our surprise, whereas other MSP3 family members (MSP3, MSP6, H101, and MSP11) and DBL-containing merozoite surface proteins (EBA140, EBA175, and EBA181) reacted, as expected, with only the exposed sera, DBLMSP and DBLMSP2 showed equally strong immunoreactivity to both the unexposed control and exposed sera (Fig. 1*B*). This suggested that recombinant DBLMSP and DBLMSP2 bound immunoglobulins present in normal serum from individuals without prior exposure to the malaria parasite. Indeed, when each protein was exposed to purified human immunoglobulin isotypes, strong binding was observed to IgM, but not to IgA, IgE, or IgG (Fig. 1*C*). This binding was specific to human IgM because they did not bind immunoglobulins from other mammalian species including goat, rabbit, guinea pig, and cow, or purified mouse IgM (Fig. 1*D*). To confirm these observations, we showed that DBLMSP-coated beads but not control beads incubated in normal human serum purified bands with masses there were consistent with the heavy and light chain of IgM (Fig. 1*E*), and their identities were subsequently confirmed by mass spectrometry.

IgM adopts a flat, planar structure in solution but can change to a “staple” conformation upon antigen binding, thereby permitting interaction with other proteins such as C1q (21). To determine whether antigen bound to IgM influenced its interaction with DBLMSP and DBLMSP2, we made use of two transfectomas producing chimeric IgMs that recognize dinitrophenyl but differ by either the presence or absence of a murine J-chain. In these chimeric IgMs, the whole κ light chain and the variable region of the heavy chain are of murine origin, whereas the heavy chain Fc constant region is human (22). DBLMSP and DBLMSP2 bound IgM from both transfectomas, demonstrating that they interact with the human IgM heavy chain constant region, and this binding was independent of whether they were engaged with antigen or not. Binding of DBLMSP2 to IgMs lacking a J-chain, however, was consistently weaker (Fig. 1*F*).

##### The DBL Domain of DBLMSP and DBLMSP2 Binds Directly and Avidly to the IgM Cμ_4_ Domain

**FIGURE 2.**
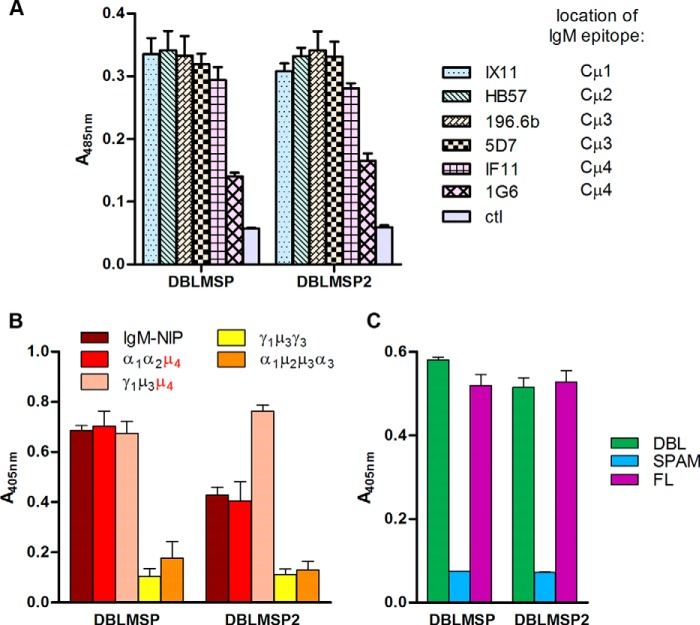
**The DBL domains directly interact with the Cμ4 domain of human IgM.** *A*, monoclonal antibodies to human IgM with known domain-specific epitopes were used to map the DBLMSP and DBLMSP2 binding domain on human IgM heavy chain. Binding of both parasite proteins was inhibited by the Cμ4-specific 1G6 antibody but not by antibodies recognizing other domains or by the IF11 antibody, which is known to bind a different epitope on Cμ4. Negative control was immobilized biotinylated IgG. *B*, DBLMSP and DBLMSP2 bound the Cμ4 domain of human IgM. Systematic testing of a small panel of chimeric human immunoglobulins containing different combinations of heavy chain domains from either IgA, IgG, and IgM (numbered and labeled α, γ, and μ, respectively) showed DBLMSP and DBLMSP2 bound the Cμ4 domain only. *C*, the DBL domain of DBLMSP and DBLMSP2 bound human IgM. Fragments encompassing the DBL, but not the SPAM domain, of both DBLMSP and DBLMSP2 bound human IgM indistinguishably from the full-length (*FL*) protein. *Bars* represent means ± S.D. in all panels; *n* = 3 (*A* and *C*) or 4 (*B*) replicate wells.

To identify the region on IgM that bound DBLMSP and DBLMSP2, we used mouse monoclonal antibodies that bind to specific domains of the human μ chain constant region (23, 24). Antibody 1G6, whose epitope is located within the Cμ_4_ domain, inhibited the interaction of DBLMSP and DBLMSP2 with human IgM, whereas antibodies IX11 and HB57, with epitopes located in the Cμ_1_ and Cμ_2_ domains, respectively, or the Cμ_3_-specific antibodies 196.6b and 5D7 did not (Fig. 2*A*). To confirm these observations, we also made use of chimeric recombinant antibodies in which domains of the human μ chain constant region replace domains from the human α or γ chain (25, 26). In all cases, binding of DBLMSP and DBLMSP2 proteins was observed only when the Cμ_4_ domain was present in the chimeric antibodies, and no binding was detected with domain-swapped antibodies containing only the Cμ_2_ or Cμ_3_ domains (Fig. 2*B*).

To determine which regions of DBLMSP and DBLMSP2 were involved in IgM binding, we individually expressed either the DBL domain or the SPAM fragment. The SPAM fragments did not bind IgM, whereas the isolated DBL domains bound IgM as efficiently as the full-length proteins in this assay, indicating that the DBL domain encompassed all IgM binding activity (Fig. 2*C*).

##### DBLMSP and DBLMSP2 Form Multimers That Interact Avidly with Human IgM

**FIGURE 3.**
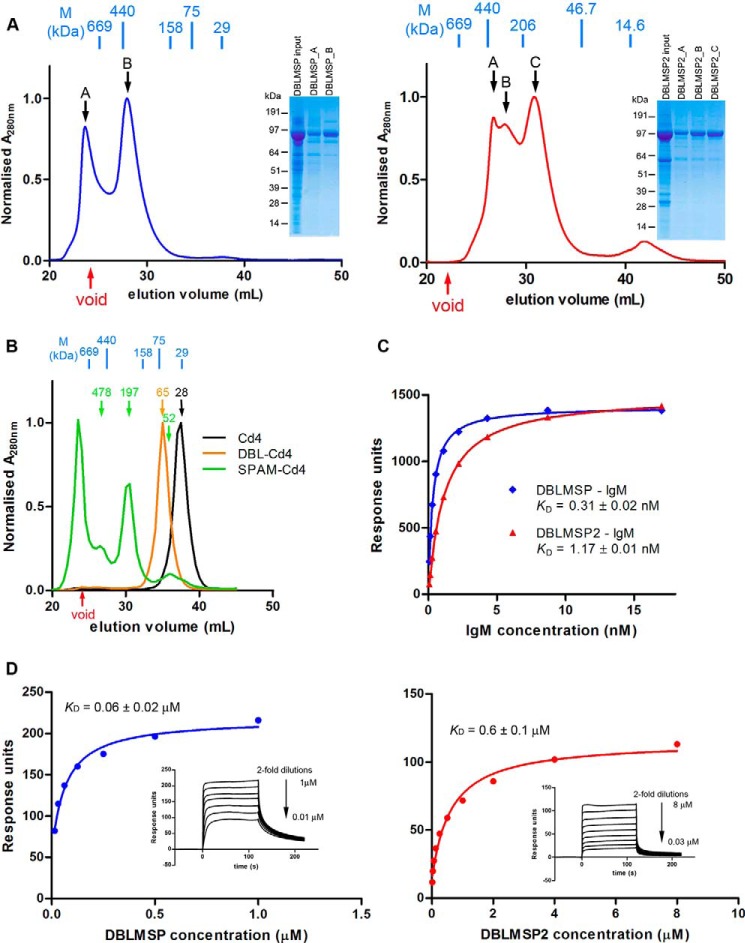
**DBLMSP and DBLMSP2 form oligomers and their DBL domain bind to human IgM with high affinity.** *A*, size exclusion chromatography showing polydisperse profile of full-length DBLMSP (*left panel*, *blue*) and DBLMSP2 (*right panel*, *red*). The indicated fractions were resolved by denaturing SDS-PAGE gel, and the *insets* show that each peak is largely composed of the full-length protein. *B*, the SPAM fragment of DBLMSP2 induces oligomerization. Size exclusion chromatography showed purified SPAM-Cd4 (*green*) was polydisperse, containing oligomers of higher molecular mass relative to DBL-Cd4 (*orange*) and the Cd4 tag alone (*black*). The predicted monomeric molecular mass of SPAM-Cd4 is 52 kDa: the major peak lies within the void volume of the column used, whereas the other two peaks correspond to theoretical molecular masses of ∼480 and ∼200 kDa, respectively. Molecular markers shown in *blue* are thyroglobulin (*669*), ferritin (*440*), aldolase (*158*), conalbumin (*75*), and carbonic anhydrase (*29*). *C*, DBLMSP and DBLMSP2 bind directly and avidly to human IgM. Equilibrium binding analysis of full-length DBLMSP (*blue*) and DBLMSP2 (*red*) to human IgM, as determined by surface plasmon resonance. Biotinylated DBLMSP or DBLMSP2 were immobilized on a streptavidin-coated chip, and 2-fold serial dilutions of human IgM were used as analyte until equilibrium was reached. Reference-subtracted data were plotted, and the *K_D_* values estimated by fitting to a simple Langmuir binding isotherm. *D*, surface plasmon resonance was used to quantify the biophysical parameters of the monomeric DBL domains of DBLMSP and DBLMSP2 binding to human IgM. Increasing concentrations of purified DBL domains for both DBLMSP (*blue*, *left panel*) and DBLMSP2 (*red*, *right panel*) were injected as analytes over immobilized human IgM on a sensor chip until equilibrium had been reached (see *inset*). Reference-subtracted binding data were plotted as a function of injected DBL protein concentration, and the *K_D_* values were estimated by fitting to a simple (1:1 binding) Langmuir binding isotherm. Binding of both DBL domains showed clear evidence of saturation demonstrating the specificity of the interaction. The DBL domain of DBLMSP reproducibly bound with ∼10-fold higher affinity than DBLMSP2.

To determine whether DBLMSP and DBLMSP2 could, like MSP3, form oligomers, we first used size exclusion chromatography on each full-length protein. In both cases, polydisperse peaks were observed, suggesting the formation of higher order oligomers composed exclusively of the proteins of interest (Fig. 3*A*). We then resolved purified proteins encompassing the DBL domain and SPAM fragment by size exclusion chromatography. Although the DBL domain of DBLMSP2 eluted as a single monodisperse peak consistent with it being a monomer, the SPAM fragment elution profile was polydisperse, with peaks consistent with tetrameric and other higher order complexes and only a minor fraction consistent with a monomeric form (Fig. 3*B*). A similar analysis of DBLMSP gave comparable results, suggesting that both DBLMSP and DBLMSP2 are able to form higher order oligomers by interactions through their C terminus.

To quantify the interaction between IgM and both DBLMSPs and demonstrate that the proteins interacted directly, we used surface plasmon resonance. Binding of soluble, purified human IgM was tested against immobilized full-length DBLMSP or DBLMSP2, mimicking the orientation of the proteins that would be expected *in vivo*. IgM binding to both parasite proteins was clearly saturable, demonstrating the specificity of the interactions, with equilibrium binding constants (*K_D_*) of 0.3 and 1.1 nm for DBLMSP and DBLMSP2, respectively (Fig. 3*C*). Given the multimeric nature of both IgM and the two parasite proteins studied, these measurements reflect overall binding avidity and are not easily comparable with other monomeric protein interactions. We therefore immobilized human IgM on the chip and used the monomeric DBL domains as analytes. Using this approach, the affinity for human IgM was 200–550-fold weaker, reflecting the loss of avidity but nonetheless still strong, with an equilibrium binding affinity of 60 nm for DBLMSP *versus* 600 nm for DBLMSP2 (Fig. 3*D*). The physiological concentration of IgM in human serum is ∼1–2 mg/ml (1–2 μm), which is at least 3 orders of magnitude higher than the measured binding constants and implies that all serum-exposed DBLMSP and DBLMSP2 would be rapidly and irreversibly complexed with IgM.

##### dblmsp-deficient Merozoites Invade Erythrocytes Efficiently but Do Not Bind IgM

**FIGURE 4.**
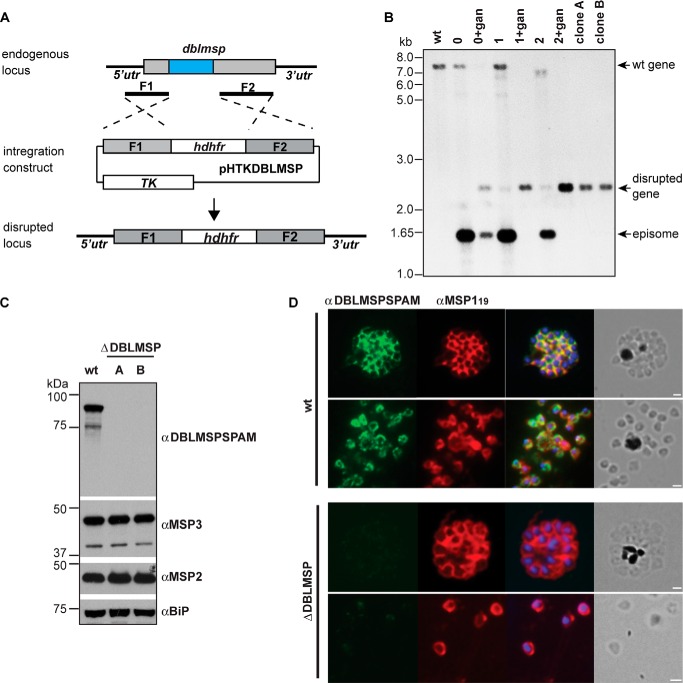
**Generation of *dblmsp* knock-out parasites.** *A*, schematic representation of knock-out strategy for *dblmsp. B*, Southern blot on EcoRV/BglII-digested genomic DNA from wild-type and transgenic parasites: genomic DNA was extracted from *P. falciparum* 3D7 wild-type parasites, transgenic parasites in presence of WR99210 with or without ganciclovir, and clonal transfectants as indicated are the *top* of the blot. Bands corresponding to the endogenous gene, episome, and disrupted gene locus are indicated with *arrows*. Size markers are given on the *left* of the panel (in kb). *C*, Western blot analysis from the lysate of 1 × 10^6^ schizonts prepared in SDS reducing sample buffer and incubated with the corresponding primary antibodies. DBLMSP could readily be detected in the lysate of wild-type parasites, but not of clones A and B. Sizes of molecular mass markers (kDa) are shown on the *left* of the panels. *D*, indirect immunofluorescence assay on thin smears of schizont and merozoites from wild-type and Δ*dblmsp* parasites showing expression of DBLMSP (*green*, *first panel*) and MSP1 (*red*, *second panel*). The *third panel* depicts the overlay of both antibodies with the nuclear stain DAPI; the *fourth panel* shows the corresponding brightfield images. *Scale bars* represent 1 μm.

**FIGURE 5.**
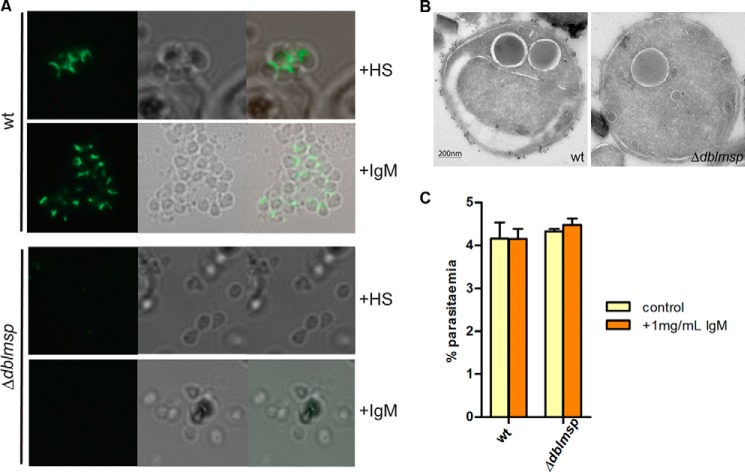
**Human IgM bind native DBLMSP protein.** *A*, indirect immunofluorescence assay for detection of human IgM was conducted on live wild-type and Δ*dblmsp* merozoites grown in either 20% human serum (+*HS*) or 0.125 mg/ml purified human IgM (+*IgM*). Alexa Fluor 488 goat anti-human IgM μ chain antibodies are depicted in *green* in *panel 1. Panel 2* corresponds to the bright field image, and *panel 3* shows the overlay. *B*, immunoelectron microscopy showing the presence of human IgM at the surface of wild-type but not Δ*dblmsp* purified merozoites. *Scale bar* represents 200 nm. *C*, addition of 1 mg/ml purified human IgM to both wild-type and Δ*dblmsp* parasites grown in Albumax did not affect their ability to infect red blood cells. *Bars* represent means ± S.D.; *n* = 3 replicate wells. A representative of four independent experiments is presented.

To demonstrate that native DBLMSP could bind human IgM, we generated Δ*dblmsp P. falciparum* merozoites in which the *dblmsp* gene has been targeted (Fig. 4*A*) and confirmed this by Southern blotting (Fig. 4*B*). Using an antibody raised against the SPAM region of DBLMSP, we showed that the Δ*dblmsp* parasites no longer expressed DBLMSP protein (Fig. 4*C*) and that it was absent from the surface of merozoites (Fig. 4*D*). We then used immunohistochemistry to test for the presence of human IgM at the surface of 3D7 wild-type or Δ*dblmsp* parasites. In wild-type parasites, DBLMSP is expressed at the surface of merozoites in all schizonts, whereas DBLMSP2 is present in only a very small subset of schizonts (1% or less) (17). Following culture in the presence of purified human IgM or with human serum, IgM could be detected readily at the surface of wild-type but not *dblmsp*-deficient merozoites (Fig. 5*A*). These observations were further confirmed by immunoelectron microscopy of purified wild-type and knock-out merozoites incubated in the presence of purified human IgM, which showed regular punctate labeling at the surface of wild-type but not Δ*dblmsp* merozoites (Fig. 5*B*). These data demonstrate that native DBLMSP binds IgM and is the sole IgM-binding protein at the surface of most merozoites. Δ*dblmsp* knock-out mutants grown in the presence of medium containing a serum alternative that does not contain human IgM did not show any invasion phenotype when compared with the wild type. To determine whether the presence of human IgM on the merozoite surface had any effect on the ability of the wild-type parasite to invade red blood cells, we also performed invasion assays with parasites grown in either serum alternative alone or serum alternative supplemented with 1 mg/ml human IgM. No difference in invasion efficiency was observed, suggesting that the presence of human IgM on the merozoite surface does not affect erythrocyte invasion *in vitro* (Fig. 5*C*).

##### dblmsp and dblmsp2 Are Dimorphic

**FIGURE 6.**
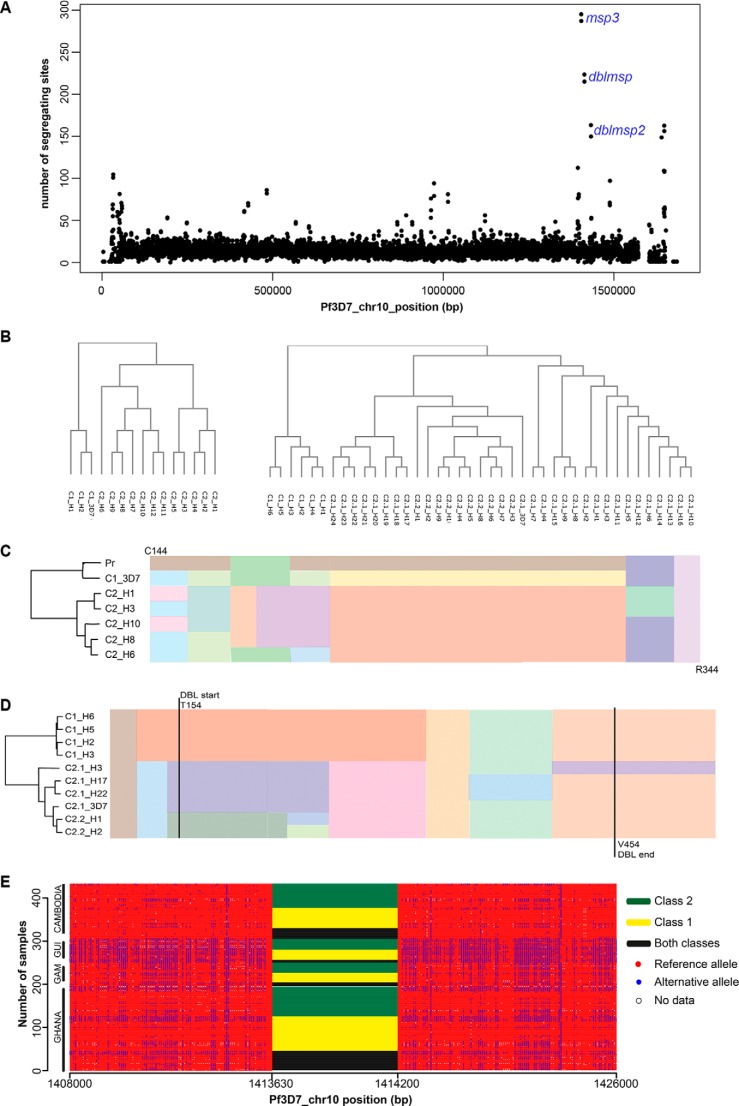
**Genome sequencing of 434 *P. falciparum* isolates reveals *dblmsp* and *dblmsp2* are dimorphic and divergent.** *A*, the number of segregating sites across chromosome 10 from 434 *P. falciparum* isolates sequenced from the Gambia, Ghana, Guinea, and Cambodia, plotted in half-overlapping 500-bp windows showing isolated regions of high sequence diversity. *B*, cluster dendrogram of the 15 *dblmsp* haplotypes (*left*) and 41 *dblmsp2* haplotypes (*right*) identified in the study. Both trees were obtained using ClustalW. *C*, a schematic representation of the amino acid multiple sequence alignment of six representative exemplar haplotypes for the DBL domain of DBLMSP between residues Cys^144^ and Arg^344^, and the *P. reichenowi* ortholog. Note the long non-recombining region has two alternate allelic forms (*orange* and *yellow*), of which the *yellow* is relatively close to the *P. reichenowi* sequence (shown in *brown*). A phylogenetic tree indicating the relationships of the sequences built by clustering haplotypes based on a distance matrix (where distance corresponds to the number of SNP and indel differences) is shown on the *left. D*, a schematic representation of the amino acid multiple sequence alignment of 10 representative exemplar haplotypes for the DBL domain of DBLMSP2 between residues Glu^102^ and Gly^550^. Note the long non-recombining region has two alternate allelic forms (*orange* for class 1 and *purple*/*dark green* for subclasses 2.1/2.2, respectively). A phylogenetic tree indicating the relationships of the sequences built by clustering haplotypes based on a distance matrix (where distance corresponds to the number of SNP and indel differences) is shown on the *left. E*, haplotype structure in the region of DBLMSP; *rows* are samples, and *columns* are polymorphic sites. Outside the DBL domain, the reference allele is shown in *red*, the alternate allele is in *blue*, and missing data are in *white*. Within the DBL domain, the class of allele found in each sample is shown: class1 only (*yellow*), class 2 only (*green*), or both present (*black*). Samples are split by country and then sorted first by DBLMSP type and then by haplotype structure outside the DBL domain. Note that the *x* axis is not drawn to scale.

Our observations suggest that the DBLMSP and DBLMSP2 proteins from the 3D7 strain of *P. falciparum* parasite are able to bind human IgM. Sequencing of some *P. falciparum* isolates from Africa has, however, shown sequence variability in *dblmsp* and *dblmsp2* (17–19). To further characterize the genetic diversity in these two genes, we looked at the sequence variability among field isolates from Africa and Southeast Asia. Characterizing and quantifying genetic architecture in field isolates using population-based genomic sequencing are challenging both because of their high degree of polymorphism and the appreciable levels of mixed infections. To circumvent these issues, we applied the Cortex *de novo* variation assembler (27, 28), which constructs a so-called de Bruijn graph representing the sequence and variation within the sample without the use of a reference genome. This method is agnostic to different types of genetic variation including indels and structural variants and is highly specific. Using this method, we generated biallelic variant calls across the *P. falciparum* genome using short-read Illumina sequence data from 434 field isolates from the Gambia, Ghana, Guinea, and Cambodia (minimum 50× coverage; mean read length, 74 bp) as reported elsewhere (17, 29, 30). Comparing genome sequences across *P. falciparum* isolates revealed striking regions of high sequence diversity, for example on chromosome 10 (Fig. 6*A*), and in particular around the MSP3 gene family. Using this approach, the highest peak was observed at *msp3* whose two diverged haplotypes, within which recombination is reduced, have been very well studied in the literature and whose divergence precedes the *P. reichenowi*/*P. falciparum* split (31); however, we also observed extremely high peaks at *dblmsp* and *dblmsp2*.

After close examination of the variant calls within *dblmsp* and *dblmsp2*, we found a large number of overlapping variants. Because Cortex only calls biallelic variants, samples with multiple infections, which contain more than two alleles at a single site, will return no value. Because few sites were multiallelic in all samples, we took the union of all sites in the 434 samples and removed redundancy; this union contained the vast majority of polymorphic sites. The overlapping alleles in *dblmsp* and *dblmsp2* were long (up to ∼1 kb) and shared sequence. As a result of this, together with the unknown multiplicity of infection and lack of phasing information with the short Illumina reads, we took a conservative approach and did not attempt to genotype the implied long multiallelic variants. Applying these approaches, we established that *dblmsp* and *dblmsp2* each contained one major cluster of long non-recombining haplotypes (15 haplotypes in *dblmsp* and 41 in *dblmsp2*), with differing lengths (∼570 bp in *dblmsp* and 1350 bp in *dblmsp2*) and all overlapping the region encoding the DBL domain. In *dblmsp*, these 15 haplotypes grouped into two major allelic forms that we termed class 1 (3 haplotypes) and 2 (12 haplotypes) (Fig. 6, *B* and *C*). In *dblmsp2*, we found two major allelic forms, class 1 (6 haplotypes) and class 2 (35 haplotypes), which we subdivided into two subclasses (c2.1 and c2.2) on the basis of multiple sequence alignments (Fig. 6, *B* and *D*). To quantify the divergence between haplotypes, we designated d(c1,c2) as the mean number of SNP and indel differences between classes c1 and c2. For *dblmsp*, d(c1,c1) = 1.77 and d(c2,c2) = 22.5, whereas d(c1,c2) = 185. Similarly for *dblmsp2*, d(c1,c1) = 2.9, d(c2.1,c2.1) = 34.2, d(c2.2,c2.2) = 7.0, and d(c2.1, c2.2) = 61, whereas d(c1, c2.1) = 239 and d(c1,c2.2) = 194, demonstrating that for both genes, the two main allelic classes were deeply diverged.

To get an estimate of population frequencies, we used the alignments to find sequence markers characteristic of the main classes and then queried the de Bruijn graph for all 434 samples to see which samples showed evidence of class 1 and/or class 2 haplotypes. Using this approach, we were able to find evidence for a haplotype from either dimorphic form even in samples where no call had been made. Our findings confirmed that both dimorphic forms co-existed at high frequencies in each gene and all four countries (Fig. 6*E*). Haplotype classes in all 434 samples were clearly identified in the region encoding the DBL domain, with SNP and indel sites present on either side. Outside the DBL domain, a strong long range linkage disequilibrium structure was observed, but all four countries showed different patterns. Within the DBL domain, however, samples from all countries showed both allelic classes at appreciable frequencies, a powerful signal of balancing selection. Although this had been shown previously in Africa, to our knowledge this is the first time it has been demonstrated in Southeast Asia. In the case of *dblmsp*, one of the two haplotype classes was very close to the *P. reichenowi* sequence (Fig. 6*C*) and is a strong example of balancing selection. Until further samples of *P. reichenowi* are sequenced, we will not know whether the selection pressure is present in *P. falciparum* only or whether this is a trans-species polymorphism (32). Interestingly, the population counts for haplotype classes in *dblmsp* showed much higher levels of mixed infection than expected (Fig. 6*E*), from which we inferred the existence of a *dblmsp* paralog, as has been suggested before (19). In conclusion, the lack of recombination within the class-defining region of the DBL domain and the long range linkage disequilibrium observed on either side of this region are striking and provide convincing evidence for strong selection pressure on the DBL domains of DBLMSP and DBLMSP2, suggesting they play an important role in parasite biology.

##### Most Naturally Occurring DBL Domain Variants Can Bind Human IgM

**FIGURE 7.**
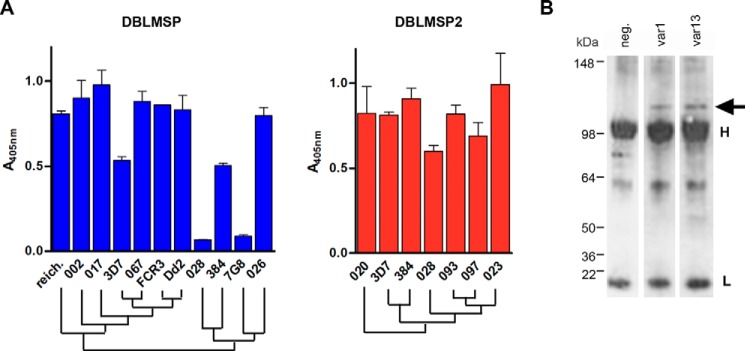
**Binding to human IgM is conserved across DBLMSP and DBLMSP2 variants.** *A*, IgM binding to representative DBL domains from 11 DBLMSP (*blue*) and seven DBLMSP2 (*red*) was conserved. Only the DBLMSP DBL domains from the laboratory strain 7G8 and isolate 028 did not show binding. A representative from two experiments is shown in each panel. *Bars* represent means ± S.D.; *n* = 3 replicate wells. *reich*, *P. reichenowi. B*, Western blot showing that DBLMSP is associated to human IgM that were purified from long term cultures of IT4 var1 or var13 parasite; the negative (*neg*) control corresponds to culture medium without parasites. Note that the anti-rabbit secondary antibody used in this experiment cross-reacts with the light (25 kDa) and heavy chains (50 kDa) of human IgG.

Because the genetic variability within *dblmsp* and *dblmsp2* is concentrated on the region encoding the DBL domain, we tested whether the ability to bind IgM was retained across DBL sequence variants representing the major allelic forms for both genes found in our and others' analyses (18, 19). For DBLMSP, a total of 10 DBL variants from 3 *P. falciparum* laboratory strains: 7G8, Dd2, and FCR3; 6 field isolates; and the *P. reichenowi* ortholog sharing between 59 and 99% sequence identity were selected (supplemental Fig. S1). Except for the DBL domains from 7G8 and 028, IgM binding was conserved across all variants, including the most divergent (384 and *P. reichenowi*) (Fig. 7*A*). Similarly for DBLMSP2, seven representative DBL variants with sequence identities ranging from 62 to 98% were selected (supplemental Fig. S1). Although variant 082 repeatedly did not produce protein, all the other six bound human IgM (Fig. 7*A*). Interestingly, whereas the 7G8 and 028 DBLMSP DBL domains could not bind human IgM, their shared DBLMSP2 DBL sequence was able to do so.

To further confirm that binding to IgM was conserved among sequence variants, we performed IgM pulldown from long term parasite culture supernatants from the var1 and var13 substrains of the IT4 strain (whose *dblmsp* sequence is identical to that of FCR3) grown in the presence of human serum. Following SDS-PAGE and incubation with an anti-DBLMSP antibody, a band corresponding to the DBLMSP protein was identified in the immunoprecipitates from parasite cultures but not from the negative control supernatant, in which no parasite was grown (Fig. 7*B*). In summary, despite their high level of sequence polymorphism, binding of the DBL domains to human IgM was conserved across most of the DBLMSP and DBLMSP2 DBL domain variants tested.

##### Binding of Human IgM to Their DBL Domains Masks DBLMSP and DBLMSP2 from Host Antibodies

**FIGURE 8.**
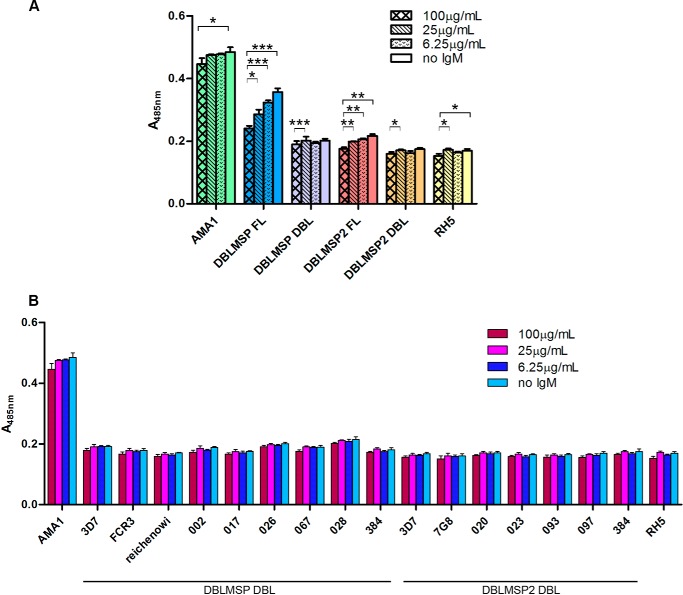
**IgM binding to full-length DBLMSP and DBLMSP2 masks them from the host immune system.** *A*, normalized amounts of full-length (*FL*) DBLMSP, DBLMSP2, RH5, the entire ectodomains of AMA1, or the DBL domain of both DBLMSP and DBLMSP2 expressed as β-lactamase-tagged pentamers were precomplexed with varying concentrations of human IgM, and the immunoreactivity of the complexes to purified IgG from Malawian adults quantified by β-lactamase substrate hydrolysis. The immunoreactivity of full-length DBLMSP and DBLMSP2 decreased with increasing concentrations of IgM. The AMA1 and RH5 proteins were used as exemplars of high and low immunoreactivity, respectively, and their immunoreactivity, as well as those of the DBL domains, was only marginally affected by the presence of IgM. *, 0.02 < *p* ≤ 0.05; **, 0.01 < *p* ≤ 0.02; ***, *p* < 0.01; two-tailed paired *t* test. Shown is one representative of three experiments. *Bars* represent means ± S.D.; *n* = 3 replicate wells; all proteins were the 3D7 strain sequences. *B*, DBL domains from a representative panel of DBLMSP and DBLMSP2 variants were expressed as β-lactamase pentamers and precomplexed with varying concentrations of human IgM before incubating with purified immobilized IgG from Malawian adults. The immunoreactivity of all the DBL domains was very low, irrespective of the IgM concentration. The entire ectodomains of AMA1 and RH5 from the 3D7 strain were used as exemplars of high and low immunoreactivity, respectively. *Bars* represent means ± S.D.; *n* = 3 replicate wells.

The fact that the DBL domains of DBLMSP and DBLMSP2 retain IgM binding despite their extraordinary sequence diversity suggests that DBLMSP and DBLMSP2 are targeted by the host immune system and that IgM binding promotes parasite survival. To determine whether individuals regularly exposed to *P. falciparum* mount an immune response to DBLMSP and DBLMSP2, we compared the immunoreactivity of the full-length proteins and DBL domains of both DBLMSP and DBLMSP2 from the 3D7 strain to purified IgG from Malawian immune adults (33). As comparators, we used AMA1 and RH5, which have previously been shown to be highly and weakly immunoreactive, respectively, to antibodies in sera from Kenyan, Malian, and Senegalese immune adults (20, 34–36). In the absence of human IgM, the immunoreactivity of the full-length 3D7 isoform of DBLMSP was intermediate, being weaker than the immunoreactivity of AMA1 but significantly higher than that of RH5. Full-length DBLMSP2, however, was only very slightly immunoreactive when compared with RH5 (Fig. 8*A*). Consistent with the large number of variants observed in *P. falciparum* populations, almost no binding of purified hyperimmune IgG was observed to either of the two DBL domains. To confirm this was not merely because the sera were collected from individuals who had not been exposed to the 3D7 variants used in this assay, we also tested the immunoreactivity of other DBL variants for DBLMSP and DBLMSP2 and confirmed that they were generally not immunoreactive (Fig. 8*B*).

Non-immune human IgM is also known to bind to some *P. falciparum* PfEMP1 variants expressed at the surface of infected erythrocytes, which are associated with rosetting or placental malaria (24, 37). Although the function of IgM binding in rosetting remains unclear, association to the var2CSA variant responsible for placental malaria has been linked to masking of the parasite from antigen-specific IgG (38, 39). To determine whether binding of IgM to the DBL domains of DBLMSP and DBLMSP2 could have a steric immunological “masking” effect, we precomplexed each of the two parasite proteins with varying concentrations of purified human IgM before incubating them with purified hyperimmune IgG. The presence of IgM significantly reduced the overall immunoreactivity of both full-length proteins in a dose-dependent manner, an effect that was not observed for AMA1 and RH5 (Fig. 8*A*). Interestingly, the presence of IgM had almost no effect on the already very limited immunoreactivity of the DBL domains. This suggests that binding of IgM to DBLMSP and DBLMSP2 masks antibody epitopes that are located in other regions of the proteins.

## Discussion

The deeply diverged lineages preserved in *dblmsp* and *dblmsp2* across *P. falciparum* populations suggests that they are under extremely strong selection and must have important biological functions. One possibility is that they are direct targets of the host immune response and are therefore potential targets for therapeutic intervention, but their function has remained unknown. Here, we have demonstrated that the DBL domains in both proteins bind directly and avidly to host IgM and that this function is conserved between the different sequence variants identified in *P. falciparum* isolates.

The population-based sequence analysis of *dblmsp* and *dblmsp2* reported here extends earlier findings that both genes exhibit a strong allelic dimorphism with limited interallelic recombination across parasite isolates from all populations sampled. Strikingly, we have now shown that both allelic classes in *dblmsp* and *dblmsp2* are present at relatively high frequency in populations from West Africa (Ghana, the Gambia, and Guinea) and Southeast Asia (Cambodia), with recombination absent (or selected against) within a specific block of the DBL domain. The pattern of balancing selection preserving two deeply diverged haplotypes with reduced recombination has previously been seen in *msp3*, which lacks a DBL domain. We would draw a distinction between this pattern, and the known dimorphism at EBA175, which is simply a single indel, rather than a long preserved haplotype. This unusual genetic architecture raises the question as to how this dimorphism arose and is maintained within the population. Following our demonstration that the DBL domain can bind host IgM, one possibility is that there is a selective advantage to binding the two major IgM species that either contain or lack a J-chain. Dimorphism, however, is also observed in genes encoding other merozoite surface proteins, suggesting selection by a broader dichotomous feature within the immune system of the host population.

Despite the large number of polymorphisms within the DBL domains of both DBLMSP and DBLMSP2, the ability to bind the constant region of human IgM was broadly conserved across representative sequence variants, suggesting that this interaction plays an important role in the parasite biology. Using the known crystal structure of the DBL domain for DBLMSP2 (9) to model the DBL domains of DBLMSP and DBLMSP2 used in our functional analysis, we observed that the majority of the differences between IgM binders and non-binders are located in helices h1, h2a, and h4 (supplemental Fig. S2). These helices could therefore be potential binding sites for human IgM. Although DBLMSP is highly expressed in all mature schizonts in the parasitophorous vacuole and located on the merozoite surface, DBLMSP2 is only expressed in a small percentage of schizonts (17, 40). One suggestion could therefore be that DBLMSP2 is expressed at the surface of the minority of merozoites committed to gametocytogenesis. One interesting observation was that although all tested DBLMSP2 variants were able to bind IgM, some DBLMSP variants did not. Although this may indicate that *dblmsp* function—or its ability to bind IgM—is dispensable, which may be consistent with an earlier finding that some *dblmsp* sequences from field isolates contained premature stop codons (18, 19), 3 of 14 cloned *P. falciparum* lines were found to contain multiple sequences for *dblmsp* (19). These results, combined with our observation of a higher proportion than expected of mixed haplotypes at the *dblmsp* locus, suggest the existence of functional paralogous *dblmsp* genes that can substitute in some isolates and strains.

Our finding that DBLMSP binds to IgM with a *K_D_* almost 4 orders of magnitude lower than physiological plasma IgM concentrations demonstrates that DBLMSP would rapidly and irreversibly be saturated with host IgM once merozoites are exposed to host blood following schizont rupture. Consequently, plasma-exposed DBLMSP should really be considered as a DBLMSP-IgM complex: something that must be taken into account when investigating the role of these proteins in the blood stages of the parasite. Previous research has suggested a role for DBLMSP in erythrocyte invasion, supported by the presence of a DBL domain, which is shared by other invasion ligands and its localization to the surface of merozoites (9, 40, 41), through its interaction with MSP1 (42, 43). However, addition of anti-DBLMSP antibodies at very high concentration (20 mg/ml) only had a modest 25% decrease on invasion (41), and deletion of the *dblmsp* gene in the 3D7 laboratory strain did not affect the invasion efficiency of the parasite in our study and others (41). The presence of human IgM in the parasite culture did not either affect the ability of wild-type or Δ*dblmsp* parasites to invade red blood cells, suggesting that DBLMSP is not essentially required for parasite invasion *in vitro*.

DBL domains, in addition to binding erythrocyte receptors from the glycophorin family, are known to mediate IgM binding in some PfEMP1 variants that are displayed on the surface of infected erythrocytes (37, 44–46). This IgM binding, which is involved in adhesion of infected erythrocytes (47, 48), has recently been suggested to cluster PfEMP1 on the erythrocyte surface thereby increasing their avidity for host receptors (49, 50). Alternatively, PfEMP1-mediated IgM binding could also be implicated in masking from immune IgG, thereby acting as a steric shield from the host adaptive immune system (38). The conservation of IgM binding to DBLMSP, despite high levels of sequence polymorphism, suggests it might be an important immune evasion strategy for the parasite. Consistent with this, a comparative immunoreactivity analysis performed with purified IgG from Malawian adults revealed that the DBL variants of DBLMSP and DBLMSP2 are poorly immunogenic and that binding to human IgM further decreases the overall immunoreactivity to the full-length proteins. This masking, however, did not seem to affect the accessibility of IgGs to other merozoite surface proteins because surface labeling of PfMSP1 from 3D7 parasites grown in the presence or absence of human IgM looked similar by immunofluorescence analysis (data not shown). Binding of host IgM to DBLMSP and DBLMSP2 might therefore mask specific domains that are important for the parasite biology on these two proteins but whose function remains unknown.

In conclusion, we have extended the genetic characterization of two *P. falciparum* genes that are under strong balancing selection and shown that the proteins both bind directly and with high affinity to host IgM and thereby shield the parasite from the host adaptive immune response. Given that both infected erythrocytes and merozoites are able to bind host IgM, IgM-binding proteins could be a general immunoprotective mechanism used by *P. falciparum* and possibly other parasites and pathogens.

## Experimental Procedures

### 

#### 

##### Ethics Statement

Use of erythrocytes and serum from human donors for *P. falciparum* culture was approved by the National Health Service Cambridgeshire 4 Research Ethics Committee. All subjects provided written informed consent. The use of animals to raise antisera was performed according to UK Home Office governmental regulations and in accordance with European directive 2010/63/EU regarding the use of laboratory animals. Research was approved by the Sanger Institute Animal Welfare and Ethical Review Board.

##### P. falciparum Population Sequence Analysis

434 samples from Gambian, Ghanaian, Guinean, and Cambodian origin sequenced by the MalariaGEN consortium underwent standard QC protocols as previously documented (29) and were sequenced to a depth of at least 50× with Illumina reads (mean read length, 74 bp) and PCR-based library preparation. Samples underwent Cortex assembly using the “Independent workflow” as previously described (28) using a *k*-mer of 31. All variants called within *dblmsp* and *dblmsp2* were collated and clustered into groups separated by at least 31 bp, revealing 15 different haplotypes overlapping the DBL domain in *dblmsp* and 41 in *dblmsp2*. The GenBank^TM^ accession numbers are KM577604–KM577618 for *dblmsp* and KM589600–KM589640 for *dblmsp2*.

##### Recombinant Protein Production

All *P. falciparum* proteins were produced recombinantly by transient transfection of HEK293E cells (51) essentially as described (20) except *Py* MSP1_19_ (52). Briefly, chemically synthesized genes were codon-optimized for mammalian expression and cloned in frame with an exogenous signal peptide (53), and potential *N*-linked glycosylation sequons were mutated to prevent inappropriate glycosylation of *Plasmodium* proteins. All recombinant proteins contain a C-terminal Cd4d3 + 4 tag (54) followed by either an enzymatically biotinylatable sequence, a His_6_ tag, or a pentamerization sequence followed by β-lactamase (55). Monobiotinylated proteins were produced by cotransfecting with a plasmid encoding secreted BirA (55, 56). Proteins were processed and purified using His-Trap purification columns (GE Healthcare) as previously described (57, 58).

##### Protein Purification from Human Serum or Parasite Culture Supernatant

Streptavidin-coated paramagnetic beads (100 μl, 1 μm diameter; Life Technologies) were saturated with 20 μg of enzymatically monobiotinylated DBLMSP or a Cd4 tag-alone control, isolated with a magnet, and washed three times with PBS before incubating with 1 ml of filtered human serum (Sigma) for one h at 4 °C. Beads were washed four times with 1 ml of PBS and eluted with 200 μl of 1% SDS. 20 μl were resolved by SDS-PAGE under reducing conditions and stained with SYPRO Orange (Sigma), and the gel image captured on a Typhoon 9400 phosphorimaging device (GE Healthcare).

Anti-human IgM agarose beads (Sigma) were incubated with long term parasite culture supernatants from the IT4 var1 and var13 strains grown in the presence of human serum or control culture medium without parasite for 1 week at 37 °C. After five washes in PBS, the beads were resuspended in loading buffer in the presence or absence of DTT, and eluates were blotted onto nitrocellulose membranes (Amersham Biosciences Protran) followed by blocking in PBS, 0.1% Tween 20, 5% nonfat milk powder) and incubated for 1 h with a rabbit anti-full-length DBLMSP antibody at a 1:100 dilution. After further washes, the membrane was incubated with an anti-rabbit HRP-conjugated IgG secondary antibody (1:1000; Sigma) and developed using 3′,3′-diaminobenzidine (DAKO) according to the manufacturer's instructions.

##### Parasite Culture and Transfection

*P. falciparum* parasites (3D7 strain) were grown *in vitro* in RPMI 1640 medium containing Albumax II or 10% human serum as described previously (59) and transfected using standard protocols (60). Transfected parasite cultures were selected with 10 nm WR99210 (kind gift of Jacobus Pharmaceuticals) and 10 μm ganciclovir (Sigma-Aldrich) and cloned by limiting dilution.

##### dblmsp Knock-out Construct Design and Characterization

For gene disruption via double homologous recombination in *P. falciparum*, a 520-bp 5′ fragment (F1 region) and a 659-bp 3′ fragment (F2 region) comprising ORF and UTR sequences of *dblmsp* were amplified using primers 348F1for (5′-AATAAACCGCGGCACATTTAATTAAGGTTGTATTTACTG-3′, SacIIsite underlined), 348F1rev (5′-TTATTTAGATCTGACATTTTTAATACCCTTACAAAAATTTTC-3′; BglII site underlined), 348F2for (5′-TTATTTATCGATAGTGTTAGGGATTCTAGTAATCTAGATCAACG-3′; ClaI site underlined), and 348F2rev (5′-TTTATTCCTAGGCATATCTTCTGTCAAACCCTTAAAAATAGTTTC-3′; AvrII site underlined). Amplification products were digested using SacII + BglII (F1 flank) and ClaI + AvrII (F2 flank) and cloned into pHTK vector (60), resulting in construct pHTKDBLMSP.

For Southern blotting, the genomic DNA from *P. falciparum* 3D7 wild-type parasites, transgenic parasites in presence of WR99210 with or without ganciclovir, and clonal transfectants were digested with EcoRV and BglII, separated on a 0.7% agarose gel, and transferred onto a nitrocellulose membrane using standard techniques. A radioactive probe was generated from the 520-bp F1 flank in construct pHTKDBLMSP using random priming (Decaprime II; Ambion). Hybridization was carried out at 62 °C overnight before stringent washes and autoradiography.

For Western blot analysis, total lysate from 1 × 10^6^ schizonts was prepared in SDS reducing sample buffer and boiled at 95 °C for 5 min. The lysates were separated on precast 10% Bis Tris NuPAGE polyacrylamide gels (Invitrogen) and transferred to nitrocellulose membranes by electroblotting. The membranes were blocked and incubated with rabbit anti-DBLMSPSPAM (1:2000), rabbit anti-MSP3 (1:2000), mouse anti-MSP2 (1:1000) (61), and rat anti-BiP antibodies (1:1000). Bound antibodies were detected with horseradish peroxidase-conjugated secondary antibodies (Bio-Rad).

For immunofluorescence analysis, thin smears of schizont and merozoites from wild-type and Δ*dblmsp* parasites were incubated with anti-DBLMSPSPAM (1:2000) and anti-MSP1_19_ (clone 1E1, 1:3000) antibodies diluted in 3% BSA/PBS and incubated for 1 h at room temperature before washes and incubation with secondary Alexa Fluor-labeled (488 and 594; Invitrogen) antibodies at 1:5000.

##### Human IgM Binding to Merozoites

To detect IgM binding to merozoites, parasites were tightly synchronized before late stage schizonts were purified using 70% Percoll, put back into culture containing RPMI + Albumax II with the addition of 20% human serum or 0.125 mg/ml purified human IgM (Sigma). IgM was detected using an Alexa Fluor 488 goat anti-human IgM μ chain antibody (Molecular Probes; preadsorbed against human IgG) at 1:1000. The slides were viewed on a Zeiss Axioplan 2 imaging system with Plan Apochromat 100×/1.4 oil immersion objective. Images were captured using Axiovision 4.6.3 software and edited using Adobe Photoshop.

For immunogold labeling, merozoites were fixed in 4% paraformaldehyde in 0.1 m phosphate buffer at pH 7.4 for 1 h at room temperature, rinsed three times in buffer, and infiltrated with 1% and then 10% gelatin before immersing in 2.3 m sucrose in phosphate buffer overnight at 4 °C for cryoprotection. Frozen samples were prepared by mounting onto aluminum pins and rapidly immersing in liquid nitrogen in preparation for ultrathin 80 nm sectioning on a Leica EM FC6 ultramicrotome. Ultra thin sections were labeled as per Tokuyasu (62), with a rabbit anti-human IgM antiserum (Abcam) diluted 1:25, and detected with 10-nm protein A gold. Imaging was performed on an FEI 120kV Spirit Biotwin with a Tietz F4.15 CCD camera.

##### Cell Culture of Transfectomas Secreting Anti-dinitrophenyl Human IgM

Transfectomas expressing anti-dinitrophenyl IgM antibodies containing the human constant heavy chains either with (Xp) or without (Gp) the mouse J-chain (22) were a kind gift of Prof. Marc Shulman. Transfectomas were grown in DMEM supplemented with 10% fetal bovine serum, 0.6 mg/ml G418, and 0.02% β-mercaptoethanol, and supernatants were harvested and filtered before use in ELISA and AVEXIS experiments.

##### ELISA and Primary Antibodies

Biotinylated recombinant *P. falciparum* proteins were normalized and immobilized on streptavidin-coated plates, preblocked with HBS and 0.1% Tween 20 (HBST), 2% BSA for 30 min. After washing with HBST, serial dilutions of either pooled human sera from 10 malaria-exposed or malaria-naïve individuals, transfectoma cell culture supernatants, or purified primary antibodies were incubated for 90 min. The plates were again washed with HBST, and appropriate alkaline phosphatase-conjugated secondary antibodies were incubated for 1 h. The plates were washed with HBST and once with HBS before adding *p-*nitrophenyl at 1 mg/ml, and absorbance at 405 nm was quantified on either a PHERAstar Plus or FLUOstar Optima plate reader (BMG Labtech). For domain mapping experiments involving chimeric human antibodies, an HRP-conjugated anti-human secondary antibody was used. Following PBS washes, tetramethylbenzidine substrate was added, and absorbance was quantified at 450 nm. All procedures were performed at room temperature. Primary antibodies used were: anti-Cd4 OX68 (1:1000; AbD Serotec); purified human IgA, IgE, IgM (3 μg/ml; Sigma), or IgG (3 μg/ml; Bethyl Laboratories); human, goat, rabbit, guinea pig, or bovine serum (1:1000; Sigma); purified mouse IgM (10 μg/ml MEM-150; Abcam); or domain-swapped human antibodies at 25 nm (25, 26). All alkaline-phosphatase-conjugated secondary antibodies were from Sigma, except anti-rabbit immunoglobulins (Jackson ImmunoResearch).

##### Mapping DBLMSP and DBLMSP2 Binding Site on Human IgM

Domain-specific monoclonal antibodies that bind the human constant μ chain (23) were incubated for 90 min with either biotinylated human IgM or biotinylated human IgG (used as a control) before washing and adding pentamerized, β-lactamase-tagged DBLMSP or DBLMSP2 and incubating for a further hour. After washes, the β-lactamase substrate nitrocefin was added at 125 μg/ml, and colorimetric turnover was measured by absorbance reading at 485 nm. Antibodies were biotinylated using EZ-link Sulfo-NHS-LC-biotin (Pierce). Construction of the homology model for the DBL domain of DBLMSP and DBLMSP2 was done using the Phyre2 engine.

##### Surface Plasmon Resonance Analysis

Surface plasmon resonance analysis was performed on a BIAcore T100 instrument at 37 °C in HBS-EP buffer, using streptavidin-coated sensor chips (GE Healthcare) essentially as described (63). Briefly, 300 response units of the biotinylated Cd4 tag was used as a reference, and molar equivalents of full-length biotinylated DBLMSP or DBLMSP2 were immobilized in query flow cells. Increasing concentrations of purified human IgM were injected for 30 min at 10 μl/min until equilibrium had been reached, and binding was quantified from reference-subtracted sensorgrams. The surface was regenerated after each cycle with either 2 m NaCl for 60 s for DBLMSP, or 10 mm glycine HCl, pH 3.0, for 20 s for DBLMSP2, and duplicate injections of the same concentration of IgM in each experiment showed no loss of activity between each cycle. For the binding analysis using the DBL domains only, 500 response units of biotinylated human IgG were immobilized as a reference, and a molar equivalent of biotinylated human IgM was immobilized in the query flow cell. Each purified DBL domain was resolved by gel filtration on a Superdex 200 Tricorn 10/600 column to remove any protein aggregates that might interfere with the kinetic measurements. Increasing concentrations of DBL domains were injected at 20 μl/min for 2 min in each cycle until equilibrium had been reached. The surface was regenerated after each cycle with 2 m NaCl for 60 s, with no loss of activity. Data analysis was performed using BIAcore analysis software.

##### Generation of Polyclonal Antibodies

A polyclonal antibody against the SPAM fragment of DBLMSP was generated. Briefly, a 619-bp DNA product (corresponding to amino acids 439–633 of DBLMSP) was amplified from 3D7 genomic DNA using primers 348ForPET30 (5′-GGTATTGAGGGTCGCAAAGAATTTAAAGATAACGTTACACTTCTTAAAGC-3′) and 348revbPET30 (5′-AGAGGAGAGTTAGAGCCTTAACTTTGTTGTTGATTACTGAGTTCTTTTTCC-3′) and cloned into pET-30 Xa/LIC expression vector (Novagen). Expression plasmids were sequenced before transformation into *Escherichia coli BL21*(*DE3*)*pLysS*, and expression was induced with 1 mm isopropyl β-d-thiogalactopyranoside for 4 h at 30 °C. Recombinant DBLMSPSPAM protein was purified using nickel-nitrilotriacetic acid affinity chromatography under non-denaturing conditions. Purified rDBLMSPSPAM was used for antibody production in rabbits (Harlan Laboratories Inc.) following standard procedures. Rabbit antibodies were subsequently affinity-purified using rDBLMSPSPAM coupled to CNBr-activated Sepharose 4B (GE Healthcare) and IgG-selected using protein G-Sepharose (Sigma-Aldrich).

Polyclonal antibodies against the full-length 3D7 form of DBLMSP were raised in rabbits (Cambridge Research Biochemicals). The full-length, His-tagged protein was produced in mammalian cells, purified by nickel-nitrilotriacetic acid chromatography using HisTrap column (GE Healthcare) and injected every 2 weeks over an 11-week period with the first injection in complete Freund's adjuvant, and the subsequent five injections in incomplete adjuvant. Rabbit antibodies were subsequently affinity-purified using HiTrap protein G columns (GE Healthcare).

##### Immunoreactivity Analysis

Pentamerized, normalized β-lactamase-tagged parasite proteins were incubated with serial dilutions of purified human IgM for 90 min before being transferred to 20 μg/ml purified IgG from Malawian adults (33) and immobilized on protein G-coated microtiter plates (Pierce). After 60 min, the plates were washed and incubated with nitrocefin at 125 μg/ml. The incubations were performed at room temperature, and absorbance was read at 485 nm as described above.

## Author Contributions

C. C. identified and characterized the interactions between human IgM and recombinant DBLMSP proteins, performed the binding and immunoreactivity studies on DBL variants, and wrote the manuscript. Z. I., S. C. M., A. M., and D. P. K. collated the samples, designed, and analyzed the data corresponding to the genetic analysis of field isolates. E. K. and A. A. H. designed, performed, and analyzed the data relating to the generation and analysis of Δ*dblmsp* and wild-type parasites. A. J. P. performed the IgM purification from human serum. G. K., D. G., and G. D. purified the merozoites and performed the immunoelectron microscopy experiments. L. Y. B. and J. C. R. performed and analyzed parasite culture experiments. S. C. M. and R. J. P. performed the mapping experiment on human IgM and the immunoprecipitation of IgM from parasite culture supernatant and built the homology model. G. J. W. conceived and coordinated the study and wrote the manuscript. All authors analyzed the results and approved the final version of the manuscript.

## Supplementary Material

Supplemental Data

## Footnotes

5 The abbreviations used are:

DBL: Duffy binding-like
EBA: erythrocyte-binding antigen
MSP: merozoite surface protein.
