# Supplementary material for: Binding of Plasmodium falciparum Merozoite Surface Proteins DBLMSP and DBLMSP2 to Human Immunoglobulin M Is Conserved among Broadly Diverged Sequence Variants
Source: J Biol Chem. 2016 May 12;291(27):14285–99. doi: 10.1074/jbc.M116.722074 (PMC4933183; doi:10.1074/jbc.M116.722074)
Supplement: Supplemental Data [file supp_291_27_14285__index.html]

Binding of Plasmodium falciparum merozoite surface proteins DBLMSP and DBLMSP2 to human immunoglobulin M is conserved amongst broadly diverged sequence variants — Binding of Plasmodium falciparum Merozoite Surface Proteins DBLMSP and DBLMSP2 to Human Immunoglobulin M Is Conserved among Broadly Diverged Sequence Variants — Human IgM Binds Broadly Diverged DBLMSP Protein Variants — Supplemental Data 

# Binding of *Plasmodium falciparum* Merozoite Surface Proteins DBLMSP and DBLMSP2 to Human Immunoglobulin M Is Conserved among Broadly Diverged Sequence Variants

## Supplemental Data

- Supplementary Figures 1 and 2 (.pdf, 1.2 MB) - Supplementary Figures 1 and 2
