# Supplementary material for: Binding of Plasmodium falciparum Merozoite Surface Proteins DBLMSP and DBLMSP2 to Human Immunoglobulin M Is Conserved among Broadly Diverged Sequence Variants
Source: J Biol Chem. 2016 May 12;291(27):14285–99. doi: 10.1074/jbc.M116.722074 (PMC4933183; doi:10.1074/jbc.M116.722074)
Supplement: Supplemental Data [file 10.1074_M116.722074_jbc.M116.722074-1.pdf]

A

DBLMSP DBL variants	DNA	Protein	Boundaries
3D7	XM_001347596	XP_001347632	S135-D435
7G8	FJ406853	ACR09793	S103-D405
Dd2	FJ406861	ACR09800	S103-D403
FCR3	FJ406860	ACR09799	S103-D403
P. reichenowi	FJ406845	ACR09785	S103-D403
Isolate 002	FJ556030	ACR49847	S103-D403
Isolate 017	FJ556042	ACR49858	S103-D403
Isolate 026	FJ556049	ACR49864	S103-D405
Isolate 067	FJ556070	ACR49882	S103-D403
Isolate 028	HM000310	ADJ58681	S103-D405
Isolate 384	HM000317	ADJ58688	S103-D405

DBLMSP2 DBL variants	DNA	Protein	Boundaries
3D7	XM_001347603	XP_001347639	T154-V454
Isolate 020	HM000330	ADI99310	H134-V436
Isolate 023	HM000332	ADI99312	S131-V430
Isolate 028	HM000334	ADI99314	T131-V431
Isolate 082	HM000336	ADI99316	S131-V431
Isolate 093	HM000339	ADI99319	T131-V430
Isolate 097	HM000340	ADI99320	T131-V430
Isolate 384	HM000390	ADI99370	S131-V431

B

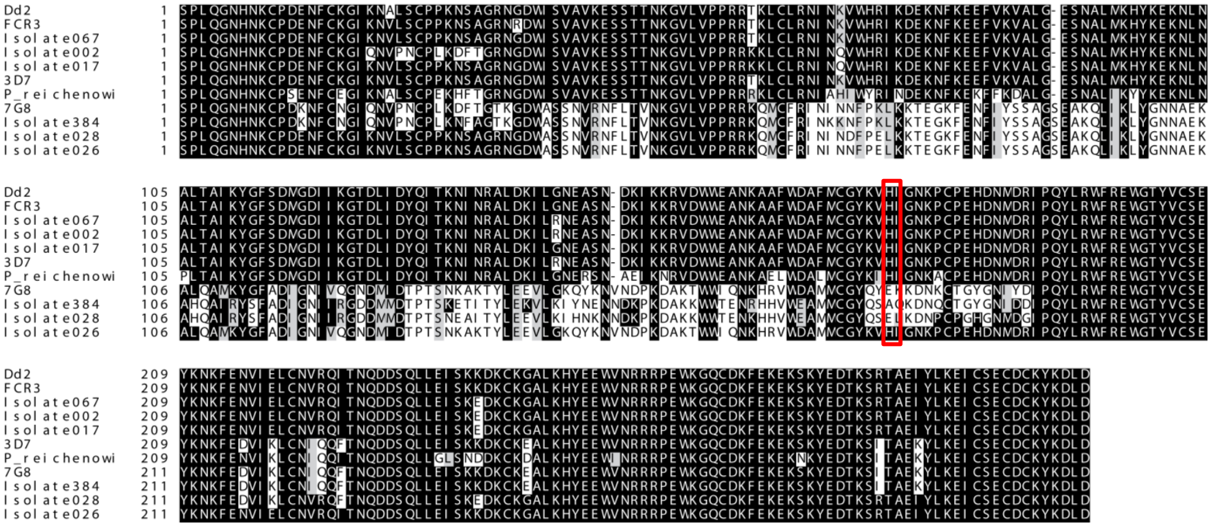

C

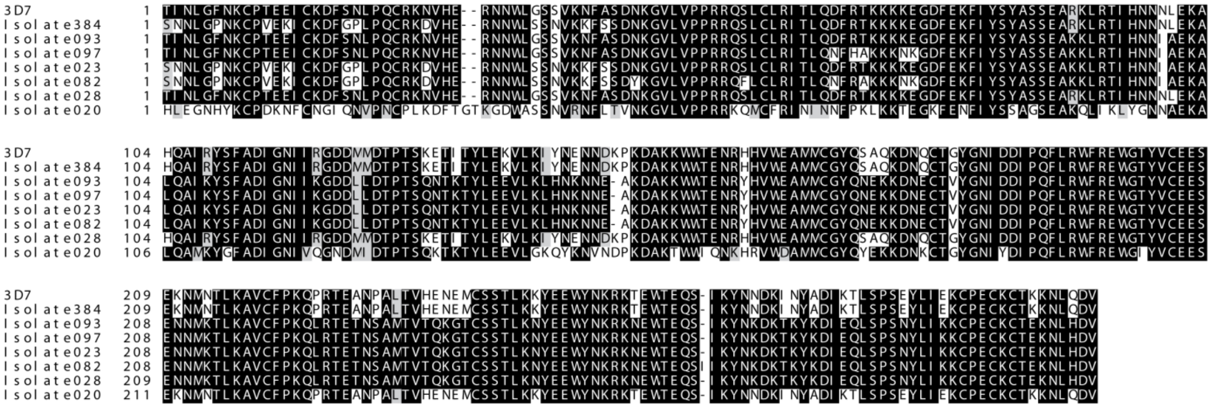

Figure S1. Details of the DBLMSP and DBLMSP2 DBL domains used for functional analysis

A. DNA and protein accession numbers of the different DBL domains used in this study. The amino-acid boundaries of each DBL domain are indicated in the right-hand column. **B.** Sequence alignment of the DBLMSP DBL domains. A common feature of the two DBL domains that do not bind IgM (7G8 and isolate 028) is the presence of a Glu amino-acid residue at position 310, where all other domains contain either a His or an Ala residue. To test whether we could narrow the binding site to IgM even further, we used site-directed mutagenesis and substituted the Glu and Leu present at positions 310-311 in the non-binding domain of isolate 028 for Ala and Gln residues present in the binding domain of isolate 384 (red box). These substitutions were however not sufficient to restore binding of the DBL domain of isolate 028 to human IgM, suggesting that these two residues are not sufficient to confer the IgM-binding property. **C.** Sequence alignment of the DBLMSP2 DBL domains. Sequence alignments of the different DBL variants were performed with ClustalW2 (http://www.ebi.ac.uk/Tools/msa/clustalw2/) and displayed using Boxshade (http://embnet.vital-it.ch/software/BOX_form.html).

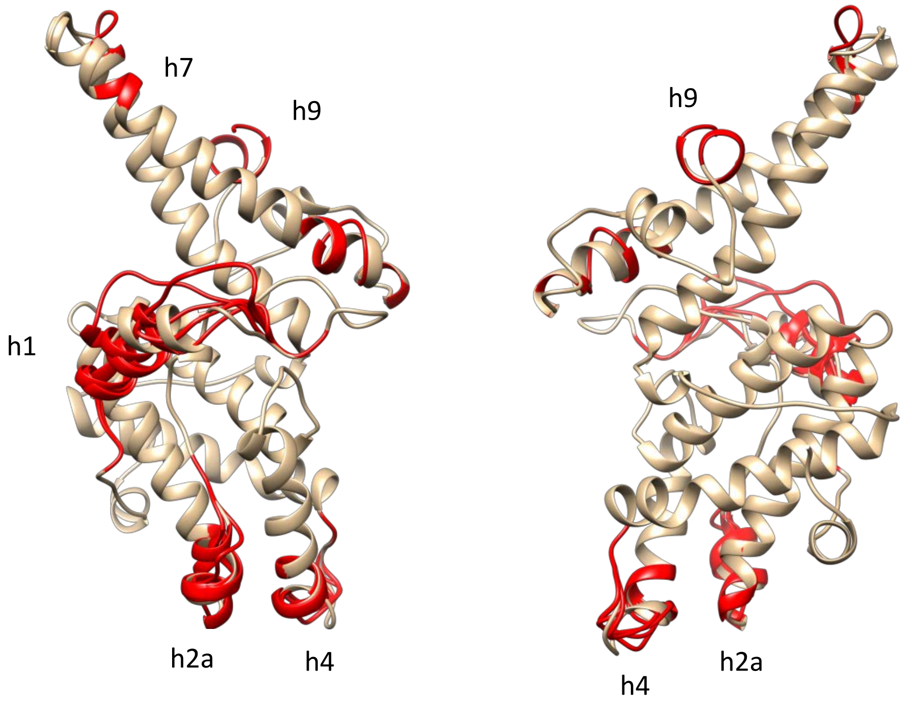

Figure S2. Modelled structural comparisons between IgM-binding and –non-binding DBL domains

The structures of the IgM-binding DBL domains from DBLMSP and DBLMSP2 were modelled using the Phyre2 software and the known crystal structure of the DBL domain from DBLMSP2. The IgM-binding DBL domain models are shown in beige, and the regions that differ in the non-binding DBL domains (isolate 028 and strain 7G8 in DBLMSP), are superimposed and highlighted in red (left panel). The right panel shows a 180° rotation of the same structure. Most differences were observed in helices h1, h2a, h4 and h7. Polymorphisms in h7 are, however, also frequently observed around the same positions amongst DBL domains that have retained their IgM-binding ability. The IgM-binding site is therefore most likely to be in helices h1, h2a or h4.
